# Blunted Microvascular Reactivity in Psoriasis Patients in the Absence of Cardiovascular Disease, as Assessed by Laser Speckle Contrast Imaging

**DOI:** 10.3390/life12111796

**Published:** 2022-11-06

**Authors:** Anastasia Margouta, Panagiota Anyfanti, Antonios Lazaridis, Barbara Nikolaidou, Konstantinos Mastrogiannis, Anastasia Malliora, Aikaterini Patsatsi, Areti Triantafyllou, Stella Douma, Michael Doumas, Eugenia Gkaliagkousi

**Affiliations:** 1 3rd Department of Internal Medicine, Papageorgiou Hospital, Aristotle University of Thessaloniki, 56429 Thessaloniki, Greece; 2 Second Medical Department, Hippokration Hospital, Aristotle University of Thessaloniki, 54642 Thessaloniki, Greece; 3 2nd Department of Dermatology and Venereology, Papageorgiou General Hospital, Medical School Aristotle University of Thessaloniki, 56429 Thessaloniki, Greece; 4 2nd Propedeutic Department of Internal Medicine, Hippokration Hospital, Aristotle University of Thessaloniki, 54642 Thessaloniki, Greece

**Keywords:** microvascular dysfunction, laser speckle contrast imaging, psoriasis, arterial stiffness, pulse wave velocity

## Abstract

Psoriasis is associated with accelerated rates of cardiovascular disease (CVD). Laser Speckle Contrast Imaging (LSCI) is a novel, non-interventional technique for the dynamic assessment of microvascular endothelial dysfunction, which represents an early precursor of CVD. We investigated whether skin microvascular reactivity is impaired in psoriasis and whether an association exists with large artery stiffening. Skin microvascular reactivity was assessed with LSCI combined with post-occlusive reactive hyperaemia protocol in psoriasis patients and controls in the absence of established CVD. Arterial stiffness and central hemodynamics were assessed throughout a whole 24 h period with the Mobil-O-Graph device. Most LSCI indices of microvascular reactivity were impaired in psoriasis patients (*n* = 90) compared to controls (*n* = 45) [baseline flux; occlusion flux; peak-to-baseline magnitude; baseline cutaneous vascular conductance (CVC); percentage increase in CVC, *p* < 0.001 for all comparisons]. In multivariate analysis, psoriatic disease predicted the above markers independently of classical CVD risk factors. Augmentation index, peripheral pulse pressure, and central systolic/diastolic blood pressure correlated with LSCI microvascular responses in the study population (*n* = 135). Pulse wave velocity significantly correlated with nearly all LSCI parameters, while the association with baseline flux was independent of CVD risk factors and psoriatic disease in multivariate analysis (beta = 0.096, *p* = 0.039). This study provides evidence of altered skin microvascular responses in psoriasis by use of LSCI, and interaction with macrovascular dysfunction, before the establishment of overt CVD. A non-interventional approach of skin microcirculation with LSCI might be used as an early indicator of vascular health in psoriasis.

## 1. Introduction

Although psoriasis has been traditionally perceived as a disease predominantly affecting the skin with cutaneous manifestations, accumulating evidence has established an association with increased rates of cardiovascular complications, including cardiovascular mortality, myocardial infarction, and stroke [1]. Epidemiological and clinical data point toward an impaired cardiometabolic profile in psoriasis characterized by the accumulation of conventional cardiovascular risk factors, which are major contributors to the establishment of cardiovascular disease (CVD) [2]. On the other hand, psoriasis potentiates systemic inflammatory responses, which have emerged as plausible pathogenetic links for psoriasis-related excess CVD risk [1,3]. As reported extensively in other immune-mediated diseases, the crosstalk between inflammation and endothelial dysfunction is the cornerstone for the development of atherosclerosis, micro- and macrovascular dysfunction, and, subsequently, clinically evident cardiovascular disease [4]. More specifically, markers of macrovascular dysfunction such as arterial stiffness and central hemodynamics, which can be monitored throughout a whole 24 h period with the newest ambulatory blood pressure (BP) devices, are strongly associated with increased rates of CVD events and death [5,6]. In addition, functional and structural alterations of the microvasculature in divergent vascular beds have been documented in patients with CVD risk factors [7,8,9,10], including chronic inflammatory conditions [11,12,13,14]. In line with the above, there is evidence for impaired vascular endothelial health in patients with psoriasis, further supporting that the study of microcirculation merits further attention to better understand the increased CVD risk in psoriasis [15].

Laser Speckle Contrast Imaging (LSCI) is a novel, non-interventional technique for the dynamic assessment of microvascular function [16,17]. In combination with reactivity procedures, including post-occlusive reactive hyperaemia (PORH), spatial and temporal reproducibility of this technique is high especially compared to conventional laser Doppler flowmetry [18,19]. LSCI has been applied as a sensitive method for revealing altered microcirculation dynamics in patients with high CVD risk, including hypertension, end-stage kidney disease, and coronary artery disease [9,20,21,22], as well as autoimmune inflammatory diseases, such as systemic sclerosis and systemic lupus erythematosus [11,23]. However, it has not been tested so far in patients with psoriasis, who are characterized by increased CVD associated with chronic inflammation and autoimmune dysregulation.

Therefore, the aim of the present study was to evaluate skin microvascular function using LSCI combined with a PORH protocol in psoriasis patients in the absence of established cardiovascular disease, as compared to non-psoriasis individuals. We further investigated the potential interactions between microvascular reactivity and markers of macrovascular function, i.e., arterial stiffness and central hemodynamics, non-invasively assessed throughout a whole 24 h period.

## 2. Methods

### 2.1. Study Participants

Consecutive patients with psoriasis were enrolled and were followed up in the Dermatology Outpatient Unit of Papageorgiou General Hospital. We included individuals matched with controls at a range of 2:1 for traditional CVD risk factors (age, hypertension, body mass index, smoking), who were thoroughly examined to exclude the presence of psoriatic lesions. The controls were recruited from the community and the Outpatient Hypertension Clinics of our department. The patients with psoriasis and controls were eligible to participate provided they were adults (>18 years old) and free from established CVD, defined as acute or past CVD events or cerebrovascular events, ischemic heart disease, heart failure, and arrhythmias. The exclusion criteria were diabetes mellitus, active infection or other than psoriasis inflammatory disorder (acute or chronic), concurrent malignancy or relapse, moderate to severe renal or hepatic dysfunction, and pregnancy. In addition, participants were excluded from the study if they presented tattoos or extensive skin burns and were menstruating at the time of examination or used oral contraceptives in the case of females. Hypertension, diabetes, and dyslipidemia were defined according to the guidelines [24,25,26]. The definition of CVD events included stroke, angina, and myocardial infarction on the basis of self-reported medical history, personal medical records, and the consideration of prescribed medication. All of the participants provided written informed consent prior to participation. The study was approved by the Aristotle University Ethics Committee and held in accordance with the Declaration of Helsinki [27].

### 2.2. Study Procedures

Demographics, anthropometric characteristics, comorbidities, and concomitant medications were recorded for every participant, and a physical examination was performed. The BMI was measured in kg/m^2^, and obesity was defined as BMI ≥ 30 kg/m^2^. Psoriasis-related parameters were additionally recorded in patients with psoriasis, specifically, disease duration and severity, which was assessed with Psoriasis Area Severity Index (PASI). Office BP was recorded with a validated oscillometric device (Microlife Exact BP, Microlife AG, Widnau, Switzerland) according to relevant recommendations [24]. All participants underwent venous blood sampling at the end of the procedures for routine biochemical measurements. The patients with psoriasis and controls were asked to abstain from food, caffeine, alcohol, and smoking for >12 h before the study visit. Then, LSCI was applied to evaluate skin microvascular perfusion.

### 2.3. Evaluation of Microvascular Perfusion with LSCI

Following an acclimatization period of 20 min with the participants lying in the supine position in a quiet, temperature-controlled room (23 ± 1 °C) with constant ambient light, LSCI (PeriCam PSI NR System, Perimed, Järfälla, Sweden) was applied according to the manufacturer’s instructions [17]. Briefly, the constant movement of red blood cells within the vessel backscatters the light that is sent by a coherent laser beam (penetration wavelength of 785 nm) toward the illuminated tissue of interest, and generates intensity fluctuations. The latter are utilized to produce a dynamic speckle pattern indicative of skin microvascular perfusion (flux), with efficient spatial and temporal resolution [28,29].

**Figure 1:**
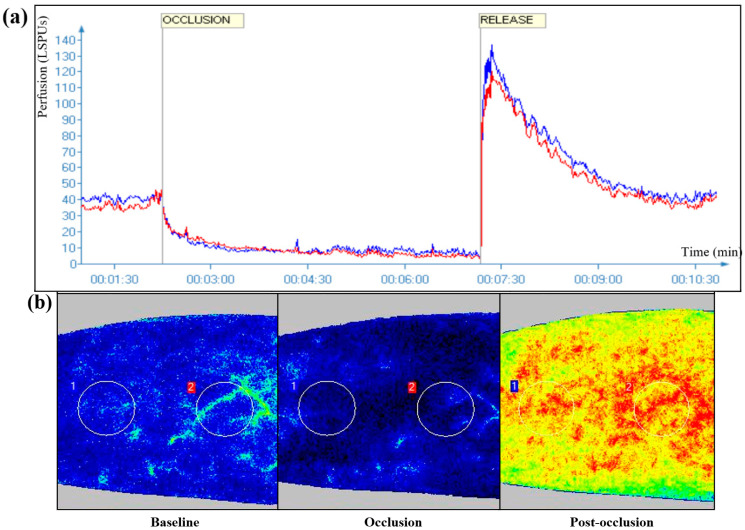
Representative laser speckle contrast imaging (LSCI) measurement with post-occlusive reactive hyperemia (PORH) protocol: (**a**) the whole recording in the selected two skin sites (circular Regions of Interest, ROIs) depicted in laser speckle perfusion units (in red and blue lines), (**b**) dynamic representation of the three recording periods (baseline, occlusion, post-occlusion) in color. The circular areas marked as “1” and “2” represent the selected ROIs. Color transition in areas from dark blue to light blue, green, yellow, orange, and, finally, red corresponds to gradually increasing laser speckle perfusion values.

LSCI was performed using a previously standardized protocol as described elsewhere [11,21,22,30]. At the beginning of the study procedure, a vacuum cushion was set below the right arm of each participant in order to eliminate moving artifacts. A sphygmomanometer cuff was placed above the elbow, and the laser head was fixed at 15 ± 1 cm from the ventral surface of the forearm to select two skin sites (circular Regions of Interest, ROIs) >10 mm^2^ [31]. A frame rate of 21 images/s with a resolution of 0.41–0.46 mm was used in the obtained images. The PORH protocol included a baseline recording period of 2 min; an occlusion period of 5 min, starting with inflation of the sphygmomanometer cuff at suprasystolic levels of 250 mmHg to obstruct blood flow, and finally, a post-occlusive period of 5 min starting with deflation of the cuff [11,16]. In this protocol, peak blood flow during the post-occlusive period coincides with the maximum skin microvascular response and subsequently returns to baseline levels. A representative recording is depicted in Figure 1. The mean blood flux of the two ROIs per testing period was calculated, with values expressed in perfusion units (PU).

The results were analyzed using the manufacturer’s software (PIMSoft, Perimed, Järfälla, Sweden). The following variables are obtained by LSCI, which correspond to skin microvascular responses: (i) baseline flux: the mean flux over the baseline period expressed in arbitrary perfusion units (Laser Speckle Perfusion Units, LSPUs), (ii) occlusion flux: the mean flux over the occlusion period (LSPUs), (iii) peak flux: the maximum flux level over the post-occlusive period (LSPUs), (iv) peak time: the time starting from cuff deflation until the moment of maximum post-occlusion flux (sec), (v) base to peak flux: the percentage increase in flux from baseline to maximum post-occlusive response (%), (vi) PORH amplitude: the difference between peak and baseline cutaneous vascular conductance (CVC), (vii) percentage increase in CVC, estimated as (PORH amplitude/baseline CVC) × 100%. The ratio of mean flux in each relevant period divided by mean BP was used to estimate CVC (in LSPUs/mmHg).

### 2.4. Assessment of Arterial Stiffness and Central Hemodynamics

The arterial stiffness indices and central hemodynamics were assessed throughout a whole 24 h period according to standard protocols using the Mobil-O-Graph (IEM), a validated oscillometric device performing ambulatory BP monitoring. The participants were instructed to follow their usual activities. The device was placed on a usual working day with a cuff of appropriate size and was set to monitor BP every 20 and 30 min over the day (0700 to 2300 h) and night (2300 to 0700 h), respectively. Daytime and nighttime periods were readjusted according to the participants’ reported sleeping hours. Only measurements with >70% valid recordings were analyzed, as recommended [24].

The Mobil-O-Graph device enables the simultaneous assessment of arterial stiffness markers and central hemodynamics, including pulse wave velocity (PWV), augmentation index (AIx), and central systolic/diastolic BP (cSBP/cDBP), which are generated from the automatic reconstruction of the aortic pulse waveform [32]. Acceptable agreement with invasive and non-invasive methods has been previously demonstrated in validation studies from both healthy volunteers and diseased populations [32,33,34]. Briefly, a high-fidelity pressure sensor (MPX5050, Freescale) records brachial pulse waves after recording brachial BP, which is then used for the calibration of the pulse waveform. To eliminate the impact of heart rate on AIx values, the automatic correction for the mean heart rate of 75 bpm was used. In addition, 24 h central pulse pressure (PP) was estimated from the difference between 24 h central systolic and diastolic BP, and 24 h peripheral PP from the difference between 24 h brachial systolic and diastolic BP.

### 2.5. Statistical Analysis

The data were analyzed with SPSS (Statistical Package for Social Sciences, SPSS Inc., Chicago, IL, USA) software, version 26. The qualitative variables were expressed as frequencies. The quantitative variables were expressed as mean ± standard deviation (SD) or median (interquartile range). The normality of the distribution was determined using the Shapiro–Wilk and Kolmogorov–Smirnov tests for samples < 50 and ≥50, respectively. The chi-square test was used for comparisons. The t-test for parametric variables and the Mann–Whitney test for nonparametric variables were used to calculate differences between the groups. Logarithmic transformation of a non-to-normal distribution was performed when necessary. Linear regression analysis with the “Enter” method was used to detect significant associations with LSCI indices while controlling for other variables. According to a recent study using LSCI in patients with rheumatoid arthritis, the estimated total sample size would be 123 individuals with a 5% level of significance, 90% power, and a sample size distribution of 1:2 for 2 groups [35]. The parametric Pearson or the nonparametric Spearman’s Rho correlation coefficient were used to calculate the correlations between the continuous variables. A probability value of *p* < 0.05 was considered statistically significant.

## 3. Results

Of the included 135 individuals, 90 were patients with psoriasis and 45 matched controls. The baseline characteristics, arterial stiffness markers, and the central and peripheral hemodynamics throughout the whole 24 h period are presented in Table 1. Although most CVD risk factors did not differ between the groups, the patients with psoriasis presented elevated total cholesterol, LDL-C, and triglycerides, and increased rates of dyslipidemia, indicative of the impaired metabolic profile associated with the disease. The 24 h central and peripheral systolic BP, PWV, and AIx were similar between the patients and controls. However, the 24 h central and peripheral diastolic BP was lower among patients, resulting in increased levels of peripheral PP, a surrogate measure of arterial stiffness. Regarding disease-related parameters, patients with psoriasis presented relatively long-term disease, and the PASI score was indicative of moderate to severe disease (Table 1).

### 3.1. Dynamic Assessment of Microcirculation with LSCI

The results from the dynamic assessment of microvascular endothelial dysfunction assessed with LSCI are presented in Table 2. Most LSCI indices were significantly impaired in psoriasis compared to non-psoriasis individuals. In particular, patients with psoriasis presented increased baseline and occlusion flux, lower peak-to-baseline magnitude, increased baseline CVC, and reduced percentage increase in CVC. Microvascular responses with LSCI in psoriasis patients and controls are further presented in Figure 1.

### 3.2. Univariate Associations of LSCI Indices with CVD Risk Factors and Disease-Related Parameters

Univariate associations were sought in the study population between LSCI indices that were significantly impaired in psoriasis patients and traditional CVD risk factors. Baseline flux significantly correlated with age (r = 0.349, *p* < 0.001), LDL-C (r = 0.194, *p* = 0.024), triglycerides (r = 0.240, *p* = 0.005), and total cholesterol (r = 0.218, *p* = 0.011), and was higher among participants with hypertension compared to their normotensive counterparts (48.8 ± 13.5 vs. 41.6 ± 11.0 LSPUs, *p* = 0.002). Occlusion flux significantly correlated with LDL-C (r = 0.202, *p* = 0.019), total cholesterol (r = 0.191, *p* = 0.027), and heart rate (r = −0.231, *p* = 0.014), and was increased in the female compared to the male participants (12.6 ± 6.8 vs. 8.5 ± 3.8 LSPUs, *p* < 0.001) and in non-smokers compared to smokers (11.6 ± 6.5 vs. 8.7 ± 3.7 LSPUs, *p* = 0.002). The peak-to-baseline magnitude was only associated with age (r = −0.267, *p* = 0.002) and was significantly lower in participants with dyslipidemia [128.9 (34.6) vs. 150.9 (62.3) LSPUs, *p* = 0.016]. Baseline CVC correlated with age (r = 0.261, *p* = 0.002), total cholesterol (r = 0.194, *p* = 0.024), and diastolic BP (r = −0.217, *p* = 0.012), and was increased among hypertensive compared to normotensive individuals (0.52 ± 0.13 vs. 0.45 ± 0.13 LSPUs/mmHg, *p* = 0.010). Finally, the percentage increase in CVC only correlated with age (r = −0.295, *p* = 0.001).

Regarding disease-related parameters, disease duration significantly correlated with occlusion flux (r = 0.287, *p* = 0.022), whereas the PASI score was non-significantly associated with LSCI indices. Subanalyis, according to treatment with methotrexate, rendered non-significant differences in all LSCI parameters.

### 3.3. Univariate Associations of LSCI Indices with 24 h BP, Arterial Stiffness and Central Hemodynamics

In the study population, significant associations were observed between PWV obtained throughout a whole 24 h period and the majority of LSCI indices, including baseline and occlusion flux, peak magnitude, peak to baseline magnitude, baseline CVC and percentage increase in CVC. These are further presented in Table 3. 24 h AIx was only associated with occlusion flux (r = 0.217, *p* = 0.014); 24 h peripheral PP only with baseline perfusion (r = 0.216, *p* = 0.014), whereas 24 h central PP was not significantly associated with skin microvascular reactivity.

Furthermore, 24 h cSBP significantly correlated with baseline CVC (−0.310, *p* = 0.001) and peak CVC (r = −0.322, *p* < 0.001), and similar associations with baseline CVC (r = −0.219, *p* = 0.013) and peak CVC (r = −0.220, *p* = 0.013) were found for 24 h brachial SBP. The 24 h cDBP significantly correlated with baseline CVC (r = −0.387, *p* < 0.001) and peak CVC (r = −0.342, *p* < 0.001), as well as PORH amplitude (r = −0.196, *p* = 0.035). Similarly, 24 h brachial DBP correlated with baseline CVC (r = −0.407, *p* < 0.001), peak CVC (r = −0.289, *p* = 0.001), PORH amplitude (r = −0.207, *p* = 0.020), and furthermore with the percentage increase in CVC (r = 0.207, *p* = 0.020).

### 3.4. Multivariate Analysis

Linear regression analysis was performed in the study population for LSCI parameters that were significantly impaired in psoriasis patients to adjust further for classical CVD risk factors and the presence of the disease. In all regression analysis models that were performed accounting for the above covariates, psoriasis remained an independent predictor of impaired microvascular reactivity, as presented in Table 4.

Lastly, multivariate analysis for 24 h PWV was performed to investigate whether univariate associations with LSCI parameters (baseline and occlusion flux, peak magnitude, peak to baseline magnitude, baseline CVC and percentage increase in CVC as presented in Table 3) would remain statistically significant. Following adjustment for psoriatic disease and classical CVD risk factors that are known to exert a significant impact on arterial stiffness, baseline flux was identified as an independent predictor of PWV (beta = 0.096, *p* = 0.039), as presented in Table 5.

## 4. Discussion

Our study has applied LSCI for the first time to assess cutaneous microvascular reactivity in healthy skin as an index of peripheral microangiopathy in patients with psoriasis. We were able to provide evidence of impaired microvascular health both at baseline recordings and during reperfusion following ischemic stimulus in a large cohort of psoriasis patients in the absence of established CVD and presumably long before the establishment of overt CVD manifestations. Furthermore, we showed that microvascular endothelial dysfunction correlates with large artery stiffening, which has been acknowledged as a surrogate marker of CVD, further supporting a cross-talk between micro- and macrocirculation in the pathogenesis of CVD. These findings imply a potential role for microcirculation dynamics as an early and sensitive CVD risk indicator in psoriasis.

Psoriasis is associated with increased rates of atherosclerotic CVD events [1]. Microvascular endothelial dysfunction is considered a hallmark of the development of CVD [36]. Psoriasis patients present with an increased prevalence of traditional CVD risk factors and impaired metabolic profile, which trigger and potentiate endothelial dysfunction [15]. In addition, the term “psoriatic march” has been introduced to describe the cutaneous-to-systemic extension of the inflammation, which activates endothelial cells both directly and indirectly and impairs their function [37]. Nevertheless, the in vivo assessment of endothelial function in psoriasis has only recently emerged as a topic of growing interest. Previous studies have documented impaired endothelial function in psoriasis patients using flow-mediated dilation (FMD) of the brachial artery [38], increased intima-media thickness of the epiaortic vessels by use of epiaortic color Doppler ultrasound and computed tomography angiography [39], and accelerated arterial inflammation with 18F-fluorodeoxyglucose positron emission tomography imaging [40,41]. However, the above-mentioned vascular techniques for the evaluation of endothelial function are either static, estimate macrovascular endothelial dysfunction, or are significantly limited by radiation exposure, reproducibility, and cost. By contrast, laser Doppler flowmetry and imaging (LDF/LDI) overcome several of these limitations by providing a dynamic, real-time evaluation of microvascular endothelial dysfunction.

To date, laser Doppler techniques have been used in psoriasis to assess vascular perfusion within psoriatic plaques [42]. However, in a small study of nine patients and an equal number of controls, nitric oxide-dependent vasodilation evaluated by LDF was attenuated in psoriasis patients, and correlated with the degree of psoriatic symptomatology [43]. Studies among patients with systemic sclerosis (SSc), one of the most studied vasculopathic diseases, offer more insight into LSCI’s extensive use and have established its value as a safe-non-contact reliable instrument for the quantification of peripheral blood perfusion at the skin level. Although the ability of LASCI to predict organ involvement in patients with systemic sclerosis remains to be clarified, median peripheral blood perfusion in hand dorsum was found to predict major vascular complication and 5-year mortality of SSc patients [44]. LSCI in SSc has been further used to evaluate response to therapies, specifically intravenous treatment with the vasoactive iloprost [45]. Further studies in psoriasis are warranted to delineate the potential value of LSCI with regard to organ involvement, the prognosis of vascular complications, and response to treatments.

By generating some novel results, our study adds to the growing body of evidence supporting the critical role of microvascular endothelial dysfunction in the pathogenesis of CVD in psoriasis. Indeed, most indices of cutaneous microvascular dynamics were significantly impaired in patients with psoriasis throughout the whole LSCI examination, from baseline flux to reperfusion following induced ischemia. The former finding merits further attention. A pattern of increased baseline perfusion has been consistently described with LSCI in other populations presenting impaired microvascular reactivity, attributed to a possible compensatory effort to recruit more functional vessels [11,30,46]. It can be further hypothesized that even this increased number of recruited vessels cannot compensate for the underlying microvascular dysfunction following an ischemic stimulus, resulting in impaired microvascular reactivity at reperfusion. Importantly, psoriasis individuals did not differ in traditional CVD risk factors compared to the controls, with the only exception of lipid profile, which is typically impaired in psoriasis [2]. Still, dyslipidemia was further taken into account in a multivariate analysis that reproduced a significant impact of psoriasis on microvascular reactivity. In our study, disease severity was not associated with microvascular dynamics, while disease duration only correlated with occlusion flux. Cumulative exposure to psoriasis-related low-grade inflammation might better correlate with vascular alterations compared to a single cross-sectional assessment of disease severity, but future studies are needed to verify this hypothesis.

Lastly, one of the most important and novel findings of our study regards the observed association between skin microvascular reactivity with several indices of arterial stiffness recorded throughout a whole 24 h period. Remarkably, baseline perfusion remained an independent predictor of 24 h PWV even after adjustment for multiple CVD risk factors and psoriatic disease. Few studies have assessed arterial stiffness in psoriasis, and even fewer have investigated potential associations with endothelial dysfunction. In a previous study recruiting plaque-type psoriasis patients, PWV and AIx were not associated with levels of endothelial progenitor cells, a circulating biomarker of endothelial dysfunction [47]. However, an interaction between the micro- and macrovasculature under the influence of systemic diseases has been consistently described in other high-CVD-risk populations from the early stages of subclinical vascular injury [48,49]. Considering the incremental value of arterial stiffness as a surrogate marker of CVD [5], the observed association of arterial stiffness with microcirculation dynamics in a population such as ours further reinforces the notion that skin microcirculation is representative of systemic vasculopathy. Notably, with the exception of cDBP and peripheral PP, the distribution of most indices of arterial stiffness and central hemodynamics was similar between patients and controls. While the investigation of pathophysiological aspects was beyond the scope of our study, this observation implies that the functional assessment of the microvasculature may represent a more sensitive marker of generalized vasculopathy in psoriasis.

The strengths of our study include a solid methodological approach. Of note, LSCI with the same protocol has been successfully applied by our group in several high-CVD-risk patients, including those with essential and secondary hypertension, systemic lupus erythematosus, chronic kidney disease, and allogeneic hematopoietic cell transplantation survivors, thus providing further evidence of the sensitivity of this technique to delineate subtle alterations in microcirculation dynamics [9,11,21,22,30]. A relatively large sample of patients with psoriasis and controls was recruited with adequate matching for several traditional CVD risk factors, while further adjustment in multivariate analysis was performed to minimize the effect of these confounders. On the other hand, limitations of our study include its cross-sectional design that does not justify causality assumptions or mechanistic explanations of the main findings. Serial measurements were not available to clarify the prognostic value of skin microcirculation dynamics in psoriasis in terms of CVD-risk prediction. The study was underpowered to delineate the effects of different drug categories, including psoriasis treatments, on microcirculation dynamics. Finally, subtle biomarkers of low-grade systemic inflammation, such as high-sensitivity C-reactive protein or proinflammatory cytokines, were not assessed.

In conclusion, the present study shows for the first-time altered skin microcirculation dynamics in psoriasis by use of LSCI in patients free from established CVD. The study further suggests an interaction between microvascular and macrovascular dysfunction, presumably long before the development of overt CVD. A non-interventional and easily repeatable approach to skin microcirculation dynamics with LSCI might be used as an early indicator of general vascular health in psoriasis. Prospective studies are warranted to determine the potential role of impaired microvascular reactivity in CVD risk prognosis and monitoring in patients with psoriasis.

## Figures and Tables

**Table 1:** Baseline characteristics and cardiovascular risk factors in psoriasis patients and control individuals.

	Psoriasis Patients (*n* = 90)	Controls (*n* = 45)	*p*-Value
a. Traditional cardiovascular risk factors			
Age (years)	49.8 ± 13.5	47.9 ± 9.6	0.342
Male sex, *n* (%)	38 (42.2)	21 (46.7)	0.624
Duration of psoriasis (years)	15 (25)	NA	NA
PASI score	10 (8)	NA	NA
Office systolic BP (mmHg)	125.7 ± 11.8	121.4 ± 13.5	0.060
Office diastolic BP (mmHg)	79.2 ± 8.7	79.8 ± 13.3	0.769
Heart rate (/min)	76.6 ± 9.8	75.8 ± 9.8	0.657
Total cholesterol (mg/dL)	214.4 ± 45.3	188.6 ± 31.8	0.001
LDL-C (mg/dL)	141.7 ± 40.8	120.1 ± 27.9	<0.001
HDL-C (mg/dL)	48 (7)	49 (14)	0.433
Triglycerides (mg/dL)	98.5 (69)	92 (43)	0.007
BMI (kg/m^2^)	28.2 ± 5.1	26.9 ± 4.7	0.155
Obesity, *n* (%)	25 (27.8)	11 (24.4)	0.680
Hypertension, *n* (%)	20 (22.2)	12 (26.7)	0.567
Psoriasis treatment, *n* (%)	18 (20.0)	NA	NA
Methotrexate, *n* (%)	13 (14.4)	NA	NA
Cyclosporine, *n* (%)	5 (5.6)	NA	NA
Antihypertensive treatment, *n* (%)	18 (20.0)	7 (15.6)	0.531
RAAS inhibitors, *n* (%)	16 (17.8)	6 (13.3)	0.510
Calcium channel blockers, *n* (%)	5 (5.6)	2 (4.5)	0.805
Beta-blockers, *n* (%)	2 (2.2)	0 (0.0)	0.319
Smoking, *n* (%)	21 (23.3)	17 (37.7)	0.096
Dyslipidemia, *n* (%)	23 (25.6)	4 (8.9)	0.022
Statins, *n* (%)	17 (18.9)	2 (4.4)	0.023
b. 24 h BP, arterial stiffness and central hemodynamics			
24 h brachial systolic BP (mmHg)	121.6 ± 11.6	122.0 ± 11.3	0.831
24 h brachial diastolic BP (mmHg)	76.7 ± 9.5	80.0 ± 8.2	0.048
24 h central systolic BP (mmHg)	118.6 ± 14.2	123.6 ± 11.9	0.075
24 h central diastolic BP (mmHg)	78.4 ± 9.4	83.2 ± 10.3	0.026
24 h PWV (m/s)	7.2 ± 1.5	7.0 ± 1.1	0.279
24 h AIx (%)	25 (10)	24 (13)	0.378
24 h peripheral PP (mmHg)	44.8 ± 7.6	40.1 ± 15.1	0.019
24 h central PP (mmHg)	40.2 ± 9.9	40.5 ± 12.2	0.893

Values are presented as mean ± SD or median (interquartile range) for continuous variables, or number (percentage) for categorical variables. Abbreviations: PASI: psoriasis area severity index; NA: not applicable; BP, blood pressure; LDL-C, low-density lipoprotein cholesterol; HDL-C, high-density lipoprotein cholesterol, BMI, body mass index; PWV, pulse wave velocity; AIx, augmentation index; PP, pulse pressure.

**Table 2:** Microvascular endothelial dysfunction assessed by Laser Speckle Contrast Imaging.

	Psoriasis Patients (*n* = 90)	Controls (*n* = 45)	*p*-Value
Baseline flux (LSPUs)	45.7 (16.4)	36.2 (7.5)	<0.001
Occlusion flux (LSPUs)	11.6 (7.0)	7.4 (4.2)	<0.001
Time to peak (s)	9.5 (9)	11 (8)	0.089
Peak magnitude (LSPUs)	107.0 (40.7)	97.3 (23.3)	0.475
Peak to baseline magnitude (%)	128.9 (46.4)	179.0 (62.0)	<0.001
Baseline CVC (LSPUs/mmHg)	0.50 ± 0.13	0.39 ± 0.09	<0.001
Peak CVC (LSPUs/mmHg)	1.17 (0.51)	1.05 (0.27)	0.369
PORH amplitude (LSPUs/mmHg)	0.63 (0.31)	0.74 (0.23)	0.076
Increase in CVC (%)	128.9 (59.3)	183.0 (63.9)	<0.001

Variables are presented as mean ± SD or median (interquartile range). Abbreviations: PORH, post-occlusive reactive hyperemia; CVC, cutaneous vascular conductance.

**Table 3:** Results of univariate correlation analysis of skin microvascular perfusion with PWV in the study population (*n* = 135).

Variable	Correlation Analysis for PWV	
	Correlation Coefficient r	*p* Value
Baseline flux (LSPUs)	0.422	<0.001
Occlusion flux (LSPUs)	0.193	0.030
Time to peak (s)	−0.231	0.009
Peak magnitude (LSPUs)	0.259	0.003
Peak to baseline magnitude (%)	−0.195	0.027
Baseline CVC (LSPUs/mmHg)	0.277	0.002
Peak CVC (LSPUs/mmHg)	0.092	0.304
PORH amplitude (LSPUs/mmHg)	−0.046	0.609
Increase in CVC (%)	−0.237	0.008

Abbreviations: PORH, post-occlusive reactive hyperemia; CVC, cutaneous vascular conductance.

**Table 4:** Multiple regression analysis for microvascular endothelial dysfunction assessed with LSCI in the study population (*n* = 135).

Variable	1. Dependent Variable: Baseline Flux *p* < 0.001, R^2^ = 0.332, Adjusted R^2^ = 0.293		2. Dependent Variable: Occlusion flux *p* < 0.001, R^2^ = 0.323, Adjusted R^2^ = 0.284		3. Dependent Variable: Peak to Baseline Magnitude *p* < 0.001, R^2^ = 0.285, Adjusted R^2^ = 0.243		4. Dependent Variable: Baseline CVC, *p* < 0.001, R^2^ = 0.254, Adjusted R^2^ = 0.211		5. Dependent Variable: Increase in CVC *p* = 0.004, R^2^ = 0.156, Adjusted R^2^ = 0.107	
	Beta	*p*	Beta	*p*	Beta	*p*	Beta	*p*	Beta	*p*
Age (years)	0.269	0.002	0.110	0.204	−0.205	0.021	0.163	0.072	−0.111	0.250
Gender (female)	0.055	0.472	0.350	<0.001	−0.188	0.018	0.157	0.053	−0.147	0.087
Hypertension	0.222	0.010	0.080	0.356	0.055	0.532	0.179	0.049	−0.082	0.395
Dyslipidemia	−0.051	0.529	0.026	0.749	−0.070	0.399	−0.012	0.890	0.111	0.225
Heart rate (bpm)	−0.108	0.163	−0.253	0.001	0.028	0.721	−0.146	0.075	0.216	0.014
Smoking	−0.051	0.504	−0.155	0.046	−0.105	0.184	0.004	0.957	0.061	0.474
Psoriatic disease	−0.401	<0.001	−0.294	<0.001	0.430	<0.001	−0.376	<0.001	0.261	0.004

Abbreviations: LSCI, laser speckle contrast imaging; CVC, cutaneous vascular conductance.

**Table 5:** Multiple regression model for 24 h PWV (*n* = 135).

Dependent Variable: PWV, *p* < 0.001, R Square = 0.837, Adjusted R Square = 0.826.						
Variable	Unstandardized Coefficients		Standardized Coefficients	95% Confidence Intervals for B		*p*
	B	SD	Beta	Lower Bound	Upper Bound	
Age (years)	0.090	0.005	0.797	0.080	0.100	<0.001
Male sex	−0.023	0.110	−0.009	−0.241	0.194	0.831
Mean blood pressure (mmHg)	0.026	0.006	0.174	0.013	0.039	<0.001
Total cholesterol (mg/dL)	<0.001	0.001	0.005	−0.003	0.003	0.914
ΒΜΙ (kg/m^2^)	0.010	0.012	0.038	−0.013	0.034	0.384
Smoking (yes)	−0.118	0.117	−0.040	−0.351	0.114	0.316
Psoriatic disease (yes)	0.008	0.124	0.003	−0.237	0.253	0.947
Baseline perfusion (LSPUs)	0.011	0.005	0.096	0.001	0.022	0.039

Abbreviations: PWV, pulse wave velocity; BMI, body mass index.

## Data Availability

Data available upon reasonable request from the corresponding author.
